# The relationship between physical activity intensity and domains with cardiac autonomic modulation in adults: An observational protocol study

**DOI:** 10.1097/MD.0000000000017400

**Published:** 2019-10-11

**Authors:** William R. Tebar, Raphael M. Ritti-Dias, Bruna T. C. Saraiva, Fernanda C. S. Gil, Leandro D. Delfino, Tatiana M. M. Damato, Beatriz A. S. Aguilar, Stéfany C. B. Silva, Jorge Mota, Luiz Carlos M. Vanderlei, Diego G.D. Christofaro

**Affiliations:** a São Paulo State University (UNESP), School of Technology and Sciences, Presidente Prudente; b Universidade Nove de Julho (UNINOVE), Sao Paulo, Brazil; c Research Center on Physical Activity, Health and Leisure (CIAFEL), Faculty of Sport, University of Porto, Porto, Portugal.

**Keywords:** autonomic modulation, autonomic nervous system, heart rate variability, motor activity, physical activity

## Abstract

**Introduction:**

Cardiac autonomic modulation is an important marker of cardiovascular health and the practice of physical activity is a fundamental tool for good maintenance or increments in this marker. However, it is not clear in the literature whether different intensities and different domains of physical activity are related in the same way to cardiac autonomic modulation.

**Objectives:**

This randomized epidemiologic study will examine the relationship between cardiac autonomic modulation and different intensities and domains of physical activity in a representative sample of adults.

**Methods:**

The sample of study will consist of 252 subjects aged from 18 years and above. The sample will be randomized through census sectors in which the neighborhoods, streets, and houses will also be randomized. Cardiac autonomic modulation will be assessed by heart rate variability and the intensity (mild, moderate, intense) of physical activity will be evaluated by means of accelerometers, while the different domains of physical activity (physical activity at work, occupational activities) will be evaluated through a questionnaire. The relationship between cardiac autonomic modulation and different physical activity intensities, as well as the different domains will be analyzed by linear regression, considering a statistical significance of 5% and a 95% confidence interval. This research protocol is registered in ClinicalTrials.gov at number NCT03986879.

## Abstract 2

Supplemental Digital Content is available in the text

## 1 Introduction

Cardiac autonomic modulation has emerged as an important marker of cardiovascular health, providing early information on impairments in cardiovascular function. Disorders in the balance between the sympathetic and parasympathetic nervous system can cause a series of cardiovascular problems. Koopman et al,^[1]^ in an epidemiological study with about 800 African adults, found that low patterns of cardiac autonomic modulation were associated with higher odds of mortality.

Likewise, physical activity has been shown to improve autonomic function.^[2]^ Indeed, several studies have shown that physically active subjects present better cardiac autonomic modulation than physically inactive peers.^[3–5]^ Rennie et al^[3]^ evaluated the practice of physical activity through a questionnaire in English individuals aged 35 to 55 years-old and observed that those subjects who were classified as practicing more vigorous intensity PA presented better autonomic modulation than those located in the lower quartiles of PA intensity. Kaikkonen et al,^[4]^ in a study with 107 obese adults, observed that higher levels of physical activity assessed by means of a questionnaire were positively associated with the SDNN index in adults in Finland. However, Kang et al,^[5]^ in a study of 131 Korean male workers, observed that physical activity recorded in the study was inversely related to cardiac autonomic modulation.

However, some limitations have been pointed-out in the literature, namely the use of only one instrument (questionnaire or accelerometer) to assess physical activity. When an accelerometer is used, although it is possible to analyze the intensity, it is unlikely that the different domains of physical activity can be analyzed without additional information. In general, the different domains of physical activity are analyzed through questionnaires, namely in the work environment, leisure, and through active transport, for example. Thus, the ideal strategy is to use both types of measures of physical activity, enabling verification of which intensity and domain of physical activity tend to be better related to cardiac autonomic modulation. It is also emphasized that men and women tend to present different cardiac autonomic modulation^[6]^ and, thus, analysis stratified by sex should be considered in this relationship.

Therefore, in the present project, we will investigate the relationship between the different intensities and domains of physical activity with cardiac autonomic modulation in adults. We hypothesize that subjects who perform physical activity at higher intensities will present better cardiac autonomic modulation. Considering the different domains of physical activity, our hypothesis is that physical activity in leisure has a greater relationship with autonomic cardiac modulation as it is carried-out in the free time period when the individual is more likely to be free from work stress and/or because it is, generally, an activity which the subject enjoys practicing.

## 2 Materials and Methods

This is a cross-sectional study funded by the São Paulo Research Foundation (FAPESP, process number 2017-07321-9). The protocol registration of this research is available at ClinicalTrials.gov (NCT03986879). The study procedures will take place from November 2018 until the second semester of 2020.

### 2.1 Ethical aspects

The study was approved by the Ethical Research Committee from Sao Paulo State University – Unesp, at protocol CAAE: 72191717.9.0000.5402. All the subjects who agreed to participate will sign an Informed Consent Term – Appendix 1, with all information about the research procedures and requirements, being free of any form of charge or payment, and free to desert from research at any time.

### 2.2 Sample

The sample will include adults aged 18 years or older from the city of Santo Anastacio, located in the southeastern region of Brazil, who are not injecting any type of medicine to control heart rate. Considering that the approximate population with over 18 years of age in the city of Santo Anastacio is 16000 inhabitants (IBGE), a correlation value of r = 0.23 was adopted to calculate the sample size,^[4]^ with an 80% power and 5% alpha, which determined a minimum sample size of 147 subjects.^[7]^ However, predicting possible refusals to participate in the study and losses of the subjects and data, a further 30% was added to the sample, totaling a minimum of 192 subjects to be evaluated. Conversely, as the present study will consider age, sex, and socioeconomic condition as adjustment variables, more 20 subjects was added for each adjustment variable. Thus, the final sample estimated is 252 individuals to be evaluated. The equation below was used to calculate the sample size. 
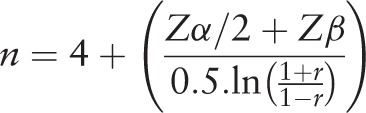


where n = sample size; Zα / 2 = alpha error value (1.96 [5%]); Zβ = beta error value (0.84 [20%]); r = correlation coefficient (Pearson or Spearman).

**Figure 1:**
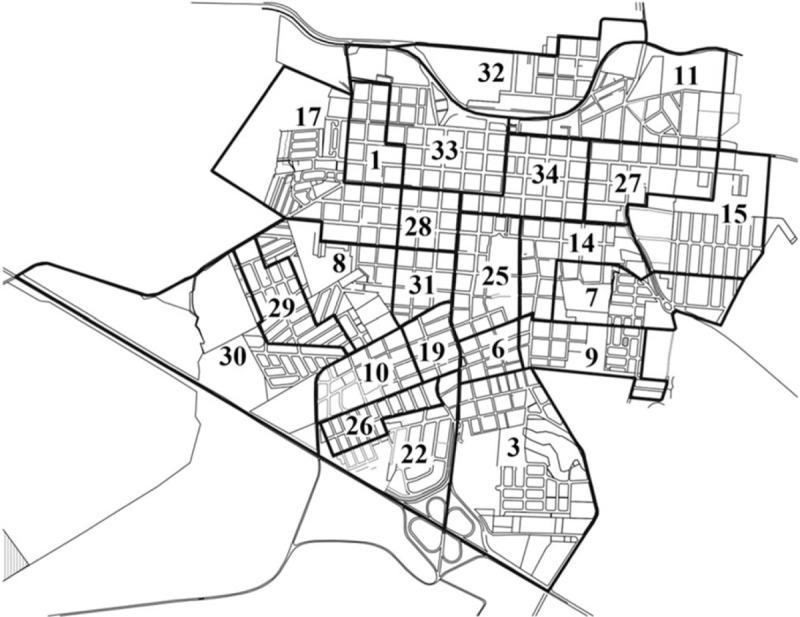
Distribution and delimitation of the urban census sectors from the city of Santo Anastacio.

For sampling, the city of Santo Anastacio will be divided according to census sectors delimitation. This city has a total of 34 census sectors, of which 23 are in the urban area of the city and will be contemplated in this study. The number of people to be interviewed in each of the census tracts will be calculated based on the number of people residing in these sectors, considering the proportional population of each census sector – Table 1. In order to carry out this procedure, the census tracts will be divided based on a geographic map of the city of Santo Anastacio – Figure 1. The neighborhoods will be registered and numbered, as well as the streets and households. Subsequently, the neighborhoods, streets, and homes will be randomly drawn based on the “random” function of SPSS. Residents of the randomly selected households aged 18 or over will be considered eligible to participate in the study and invited to participate. If the selected individuals decline to participate in the research, a new household will be randomly selected, until complete the minimum sample size required for each census sector. This sample selection design was based on the 1st Design of the National Health Survey sample.^[8]^

**Table 1:**
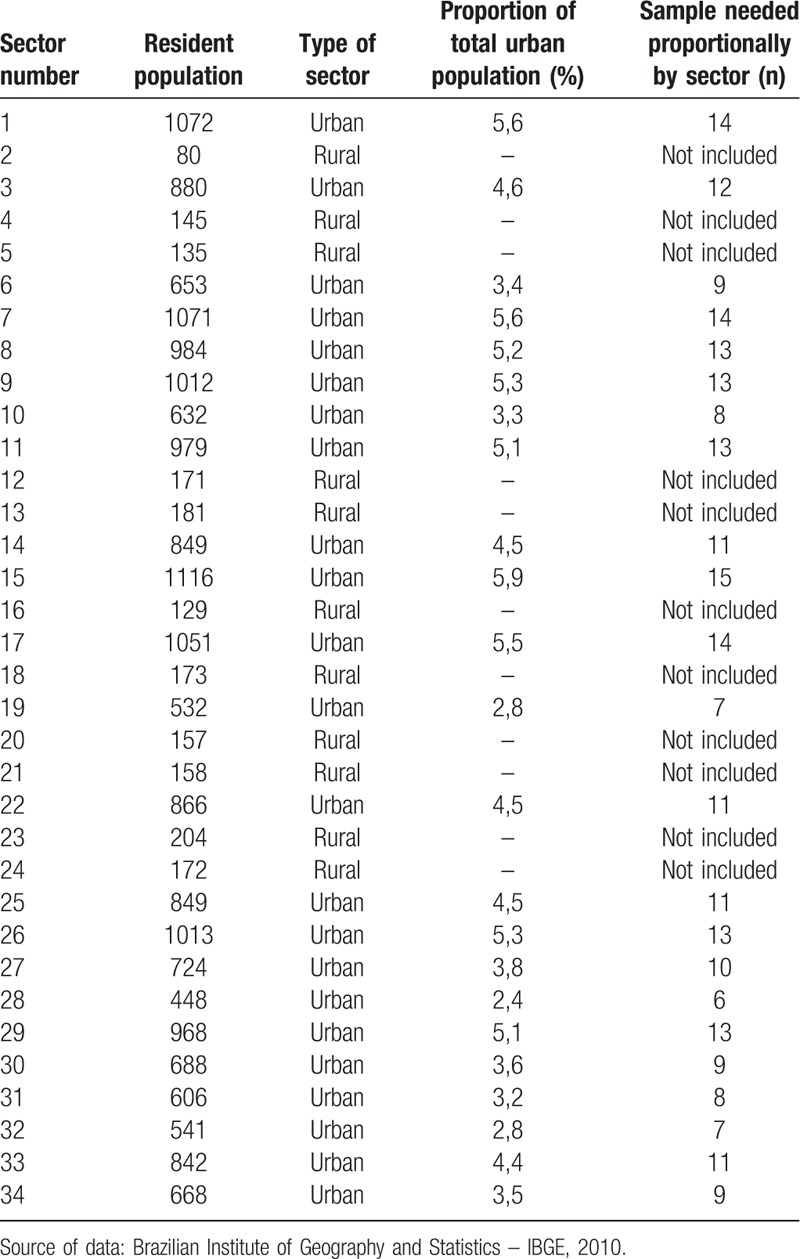
Population and proportion of sample by census sectors from the city of Santo Anastacio.

### 2.3 Data collection

**Figure 2:**
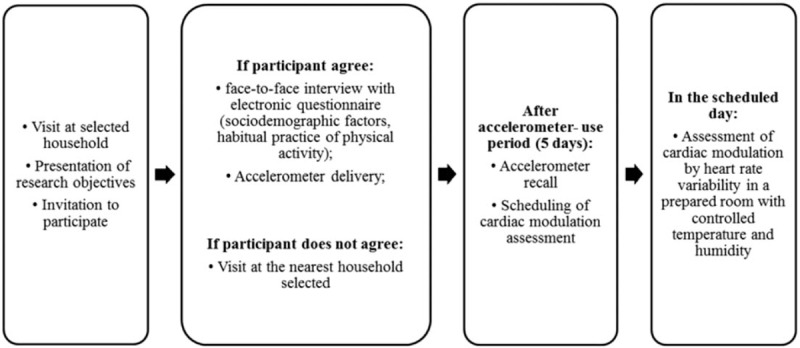
Schematic diagram of research procedures.

Initially, the evaluators will visit the census sectors drawn and the households that will be part of the sample. In this first evaluation, a face to face interview will be performed using an electronic questionnaire. The questionnaire contains physical activity domains as well as socio-demographic characteristics. After the participants have completed the questionnaires, an accelerometer will be delivered to the research participant with explanations about its use (5-day period). On a pre-scheduled date, the evaluator responsible for delivering the accelerometer will visit to the residence of the participant and perform the accelerometer recall. At that time, the evaluation of cardiac autonomic modulation through analysis of heart rate variability will be scheduled at a predetermined location, being the participant informed about the previous recommendations for this measure procedure – Figure 2.

### 2.4 Objective measurement of physical activity

Subjects will be instructed to wear the accelerometer for 5 days. Messages or phone calls will be sent/made during this period to remind the participant to wear the accelerometer. The objective measurement of physical activity will be performed using the Actigraph GT3X accelerometer (ActiGraph, LLC, Pensacola, FL) which was designed to record movements in 3 orthogonal planes: vertical, horizontal anteroposterior, and medial-lateral.

The accelerometers will be positioned laterally at waist height, and remain in position for 5 full days (3 days of the week and 2 of the weekend).^[9]^ Participants will be instructed on the care of the equipment and required to wear the equipment throughout the day (waking hours), removing the equipment only when there is contact with water (personal hygiene or any kind of aquatic activities) and while sleeping.

The ActiLife 6 program (ActiGraph, LLC, Pensacola, FL) will be used to clean the data. Each data sample, determined from counts, will be summarized considering a specific time interval, this particular interval is designated as an epoch, and lasts for 60 seconds. The 60-second period was selected because it is the closest to the pattern of low intensity and long duration activity.^[10]^ Consecutive hours of zero counts and days with less than 10 hours of monitoring will be excluded.^[11]^

To convert the raw accelerometer data to a more physiological measure, the classification according to activity intensity levels will be used. In this way, the cut-off points recommended by Sasaki et al^[12]^ for adults, performed with the same triaxial accelerometer model, will be employed in the present study. Sedentary activity will be defined as zero counts per minute, light physical activity (<3.00 METs) as less than 2690 counts per minute, moderate physical activity as counts between 2690 and 6166 (3.00–5.99 METs), vigorous physical activity as counts between 6167 and 9642 (6.00–8.99 METs), and very vigorous physical activity as values greater than 9642 counts per minute (≥9 METs).

Thus, the outcomes that will be included in this study, derived from the measurement of physical activity by the accelerometer, will be the counts per minute and total time spent in each intensity of physical activity (light, moderate, vigorous, and very vigorous).

### 2.5 Domains of physical activity

Habitual practice of physical activity in different domains will be evaluated by the Baecke et al questionnaire.^[13]^ This instrument evaluates the practice of physical activity in the previous 12 months through 16 questions, considering physical effort at work, the practice of sports activities or systematized exercises in leisure, and leisure activities. In the field of work, questions related to the physical exertion carried out at work include: time spent sitting, standing, and walking in the work environment, carrying weights, whether the evaluated subject perspires a lot at work, and how tired they feel after a day's work. In the leisure domain, the practices of physical activities during leisure are considered, such as exercises in the gym or practicing sports. In these domains, the following will be evaluated; the intensity of the physical exertion of these activities (mild, moderate, vigorous), the number of hours in the week in which these activities are practiced, and how long this physical activity has been practiced (<1 month, 1–3 months, 4–6 months, 7–9 months, > 9 months). In the field of occupational activities, the different physical activities in free time are considered, especially considering active displacement, such as the amount of time that is spent walking to the mall, market, or work; the time spent riding a bicycle is also considered.

At the end, this instrument offers a dimensionless score for each domain and the sum of the score of the 3 assessed domains determines the total amount of physical activity. This instrument has been used in different types of epidemiologic studies.^[14,15]^

### 2.6 Cardiac autonomic modulation

The evaluation of cardiac autonomic modulation will be performed through HRV. For this evaluation, subjects will be instructed not to ingest alcoholic beverages and/or stimulants, such as coffee and tea, for 12 hours prior to the experimental protocol, to avoid influences on cardiac autonomic behavior.^[16]^

For this analysis, heart rate will be recorded beat-by-beat, using an Heart Rate Monitor (Polar V800, Finland), for 30 minutes at rest in a supine position, maintaining spontaneous breathing, in a room with a temperature between 21 and 24°C and relative air humidity between 50 and 60%.^[16]^ Only circulation of the evaluators will be allowed in the room during the execution of the HRV collection.

In the series of RR intervals obtained, 1000 intervals of the most stable stroke period will be analyzed, followed by digital filtering complemented by manual filtering to eliminate ectopic, artifacts, and premature beats. Only series with more than 95% sinus beat will be included in the study.^[17]^ The HRV analysis will be performed using linear methods (time and frequency domains) and non-linear methods.

For HRV analysis using linear methods, the RMSSD (square root of the square mean of the differences between adjacent normal RR intervals in a time interval expressed in milliseconds) and SDNN (standard deviation of the mean of all normal RR intervals, expressed in miliseconds) will be used for time domain analysis.^[18]^ For the analysis of HRV in the frequency domain, the spectral components of low frequency (LF: 0.04–0.15 Hz) and high frequency (HF: 0.15–0.4 Hz) will be used, in milliseconds squared (ms^2^) and normalized units (nu), as well as the ratio between these components (LF/HF). The spectral analysis will be calculated using the fast Fourier transform algorithm.^[18]^

The Poincaré plot (qualitative and quantitative analysis [SD1, SD2, and SD1/SD2 ratio]) will be utilized to analyze Heart Rate Variability using non-linear methods. The Poincaré plot is a graphical result formed by a map of points in Cartesian coordinates.^[19]^ The qualitative analysis of the plot will be performed by means of the analysis of the figures formed by the attractor, which were described by Tulppo et al.^[20]^ The quantitative analysis will be determined by the SD1 index (standard deviation of instantaneous beat-to-beat variability), SD2 index (long-term standard deviation of continuous R-R intervals), and SD1/ SD2 ratio.^[18]^

Kubios HRV Analysis software version 2.0 (Kupio University, Finland) and Visual Recurrence Analyzes (VRA) version 4.9 (Eugene Kononov, USA) will be used for analysis of linear and nonlinear methods.

### 2.7 Socioeconomic level

The “Brazilian Economic Classification Criteria”, established in 2014 by the Brazilian Association of Research Companies,^[21]^ will be used to determine the economic condition of the participants. The questionnaire considers the degree of education, and the presence and quantity of certain rooms and domiciliary assets of the participant, and establishes the resulting classifications for economic condition: A1, B1, B2, C1, C2, and D - E. Subjects located in levels A1 and B1 will be classified as high socioeconomic class, those in classes B2, C1, and C2 as middle socioeconomic class, and those included in classes D and E as low socioeconomic class.

### 2.8 Statistics analysis

After verification of normality by Shapiro-Wilks test, the variables characterizing the sample will be expressed as mean and standard deviation or median and interquartile range. The dependent variable will be the cardiac autonomic modulation. Pearson or Spearman correlation will be used to analyze the relationship between cardiac autonomic modulation and physical activity. Linear regression will evaluate the magnitude of the relationship between cardiac autonomic modulation and physical activity, both in the unadjusted model and in models adjusted by age, schooling, ethnicity, and socioeconomic status. Confounding variables will be inserted simultaneously into the model. It should be emphasized that linear regression analysis will be performed stratifying the study participants by sex. This same type of analysis will be performed to verify the possible relationships between the different domains of physical activity (work, leisure, and displacement activity) and cardiac autonomic modulation.

## 3 Perspectives of this study

The hypothesis of the study is that different intensities and domains of physical activity will present different relationships with cardiac autonomic modulation. It is envisaged that the findings of the present study will provide better understanding about the best intensity and amount of physical activity related to the benefits in cardiac autonomic modulation. In addition, to determine if there are differences between the different domains of physical activity regarding autonomic cardiac modulation could provide guidance towards strategies of health promotion in adult population, which can be elaborated focusing on intensity and domains of physical activity.

## Author contributions

**Conceptualization:** Diego Giuliano Destro Christofaro, William Rodrigues Tebar, Raphael Mendes Ritti Dias, Luiz Carlos Marques Vanderlei, Jorge Mota.

**Data curation:** William Rodrigues Tebar, Bruna Thamyres Ciccoti Saraiva, Tatiana Machado de Mattos Damato, Beatriz Anizia dos Santos Aguilar, Stéfany Carolaine Brito da Silva.

**Formal analysis:** Diego Giuliano Destro Christófaro, William Rodrigues Tebar, Raphael Mendes Ritti Dias.

**Investigation:** William Rodrigues Tebar, Tatiana Machado de Mattos Damato, Leandro Dragueta Delfino, Bruna Thamyres Ciccoti Saraiva, Fernanda Caroline Staquecini Gil, Beatriz Anizia dos Santos Aguilar, Stéfany Carolaine Brito da Silva, Diego Giulliano, Destro Christofaro.

**Methodology:** William Rodrigues Tebar, Diego Giuliano Destro Christófaro, Tatiana Machado de Mattos Damato, Bruna Thamyres Ciccoti Saraiva, Raphael Mendes Ritti Dias, Luiz Carlos Marques Vanderlei, Jorge Mota.

**Project administration:** William Rodrigues Tebar, Tatiana Machado de Mattos Damato, Leandro Dragueta Delfino, Fernanda Caroline Staquecini Gil, Diego Giulliano Destro Christofaro.

**Supervision:** Diego Giulliano Destro Christofaro, William Rodrigues Tebar

**Writing – original draft:** William Rodrigues Tebar, Diego Giulliano Destro Christofaro.

**Writing – review & editing:** William Rodrigues Tebar, Diego Giuliano Destro Christofaro, Luiz Carlos Marques Vanderlei, Raphael Mendes Ritti Dias, Jorge Mota.

William Rodrigues Tebar orcid: 0000-0002-6192-4667.

## Supplementary Material

Supplemental Digital Content
