# Supplementary material for: The relationship between physical activity intensity and domains with cardiac autonomic modulation in adults: An observational protocol study
Source: Medicine (Baltimore). 2019 Oct 11;98(41):e17400. doi: 10.1097/MD.0000000000017400 (PMC6799861; doi:10.1097/MD.0000000000017400)
Supplement: Supplemental Digital Content [file medi-98-e17400-s001.docx]

**Appendix 1** - Informed Consent Term.

**Dear participant**

We are conducting a research project aiming to analyze the practice of physical activity, health behaviors and cardiovascular risk factors in adults from this city. In this sense, we kindly ask to sign this Informed Consent Term if you agree to participate. The research procedures corresponds to answering a questionnaire about physical activity and sociodemographic information, besides the assessment of your autonomic cardiac modulation, heart rate, anthropometric measures (body mass, height and waist circumference), blood pressure, handgrip strength and accelerometer-based physical activity. It is necessary to clarify that will be held the secrecy and privacy of your identity. It is highlighted that you have freedom to refuse to participate in the survey or desert without any type of cost or punishment. No type of charge or payment will be applied to the participants of this research. All participants will be informed about the results of evaluations. The research procedures adopted in this research are in accordance to the Ethical in Human Research Committee from National Council of Health. None of procedures adopted will offer risks about your health and dignity. At any time, you can request more information about the research through the researcher contact and through the contact of Ethical in Research Committee. After these clarifications, we request your free consent to participate in this research. Therefore, fill in the items below.

**Informed Consent Term**

I declare, in a free and informed manner, that I agree to participate in the research. I confirm that I received a copy of this term, authorizing the research procedures and the publication of the research results.

___________________________

Name of research participant

______________________________

Signature of research participant

__________________________________

Signature of researcher

**Researcher: Professor PhD Diego Giulliano Destro Christofaro. Phone: +551832295723**

**Coordinator of Ethical in Research Committee: Professor PhD Edna Maria do Carmo**

**Deputy Coordinator: Professor PhD Renata Maria Coimbra Libório**

**Committee phone: +551832295315 and +551832295526**

**E-mail:** [cep@fct.unesp.br](mailto:cep@fct.unesp.br)


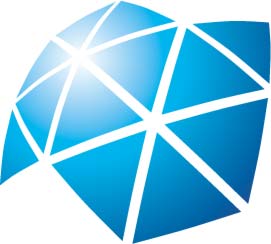
Faculdade de Ciências e Tecnologia

Seção Técnica Acadêmica

Rua Roberto Simonsen, 305 CEP 19060-900 Presidente Prudente SP

Tel 18 3229-5312 fax 18 3229-5303 sta@fct.unesp.br
